# Supplementary material for: Development of an Acid-Labile Ketal Linked Amphiphilic Block Copolymer Nanoparticles for pH-Triggered Release of Paclitaxel
Source: Polymers (Basel). 2021 May 1;13(9):1465. doi: 10.3390/polym13091465 (PMC8124141; doi:10.3390/polym13091465)
Supplement: Supplementary file 1 [file polymers-13-01465-s001.zip › polymers-1196121-supplementary.pdf]

polymers

MDPI

Supplementary Material: Degradation Studies of an Acid-Labile Ketal Linked Amphiphilic Block Copolymer Nanoparticles for pH-Triggered Release of Paclitaxel

Svetlana Lukáš Petrova *, Eliézer Jäger, Alessandro Jäger *, Anita Höcherl, Olga Janoušková, Rafał Konefał, Alexander Zhigunov, Ewa Pavlova, Martin Hrubý

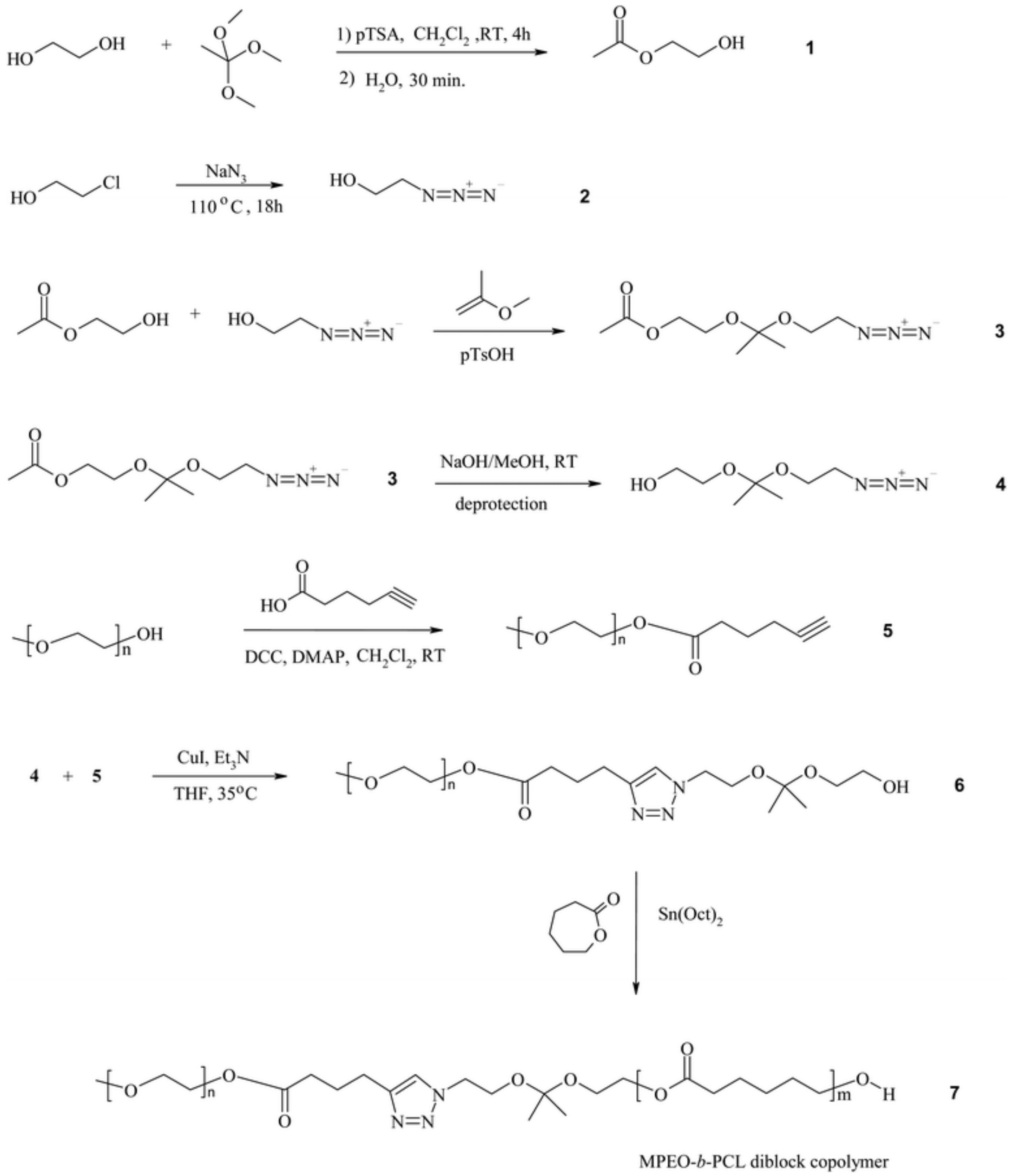

Figure S1. Synthetic route for the preparation of MPEO-*b*-PCL diblock copolymers.

Synthesis of MPEO44-*b*-PCL17 diblock copolymer containing a ketal group.

The MPEO44-*b*-PCL17 diblock copolymers were successfully synthesized by ROP from the ε-CL monomer. The previously synthesized *α*-methoxy-*ω*-hydroxy-poly(ethylene oxide) containing a ketal group (above) was used as a macroinitiator in the presence of $Sn(Oct)_2$ as catalyst. The PCL block length was controlled by regulating the ε-CL/macroinitiator molar ratio. After purification, the MPEO44-*b*-PCL17 diblock copolymer was characterised using ^{1}H and ^{13}C NMR (Figure S2), and SEC analysis (Figure S3).

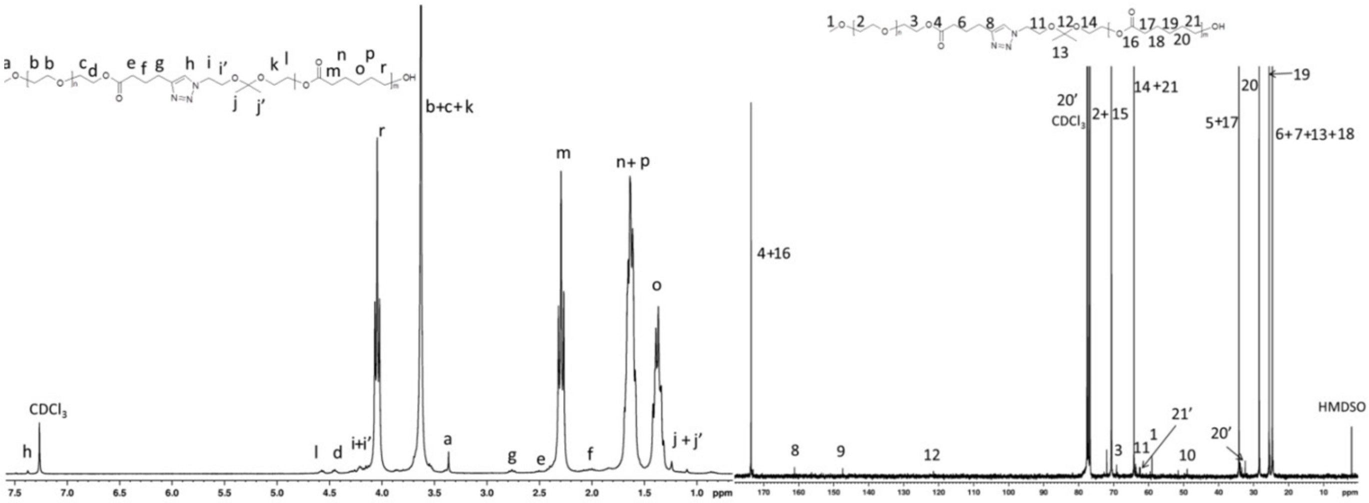

Figure S2. ^{1}H (left) and ^{13}C (right) NMR spectra of the MPEO44-*b*-PCL17 diblock copolymer in $CDCl_3$.

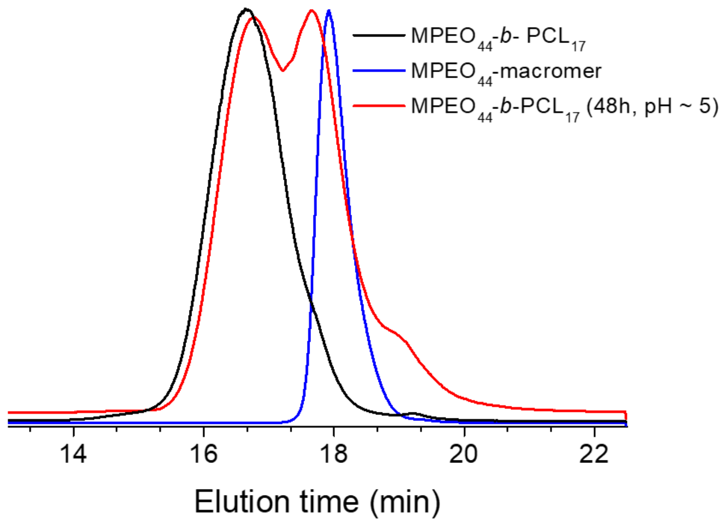

Figure S3. SEC chromatograms in THF of MPEO44-*b*-PCL17 diblock copolymer (black line), *α*-methoxy-*ω*-hydroxy-poly(ethylene oxide) macromer containing a ketal group (blue line) and the MPEO44-*b*-PCL17 diblock copolymer after degradation (phosphate buffer saline) at pH ~ 5.0 for 48h (red line).

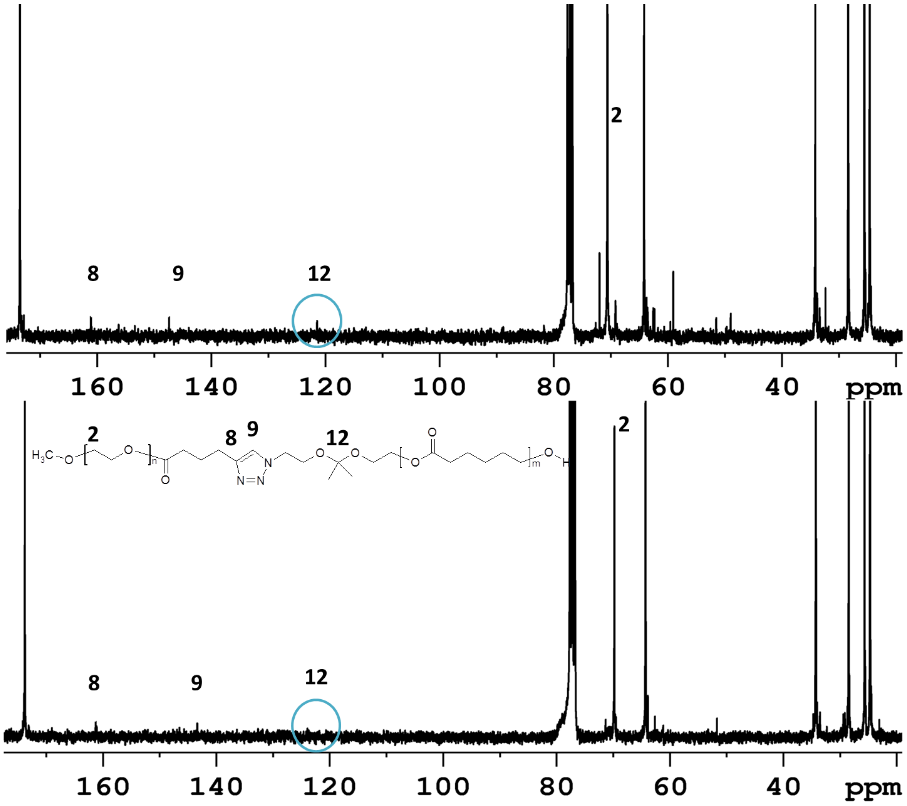

Figure S4. ^{13}C NMR spectra of $MPEO_{44}$-*b*-PCL_{17} diblock copolymer (top) before degradation and (bottom) after degradation.

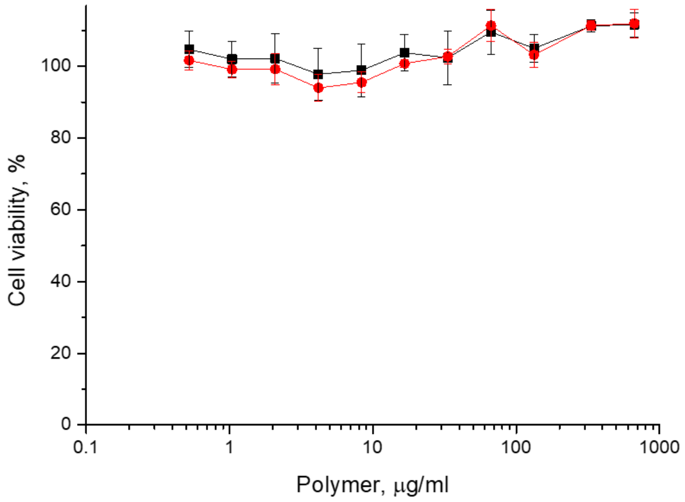

Figure S5. Cell viability of HeLa cell line after 24 h (black squares) and 48 h (red circles) incubation with different concentrations of drug-free $MPEO_{44}$-*b*-PCL_{17} diblock copolymer NPs.
